# Capacity Estimation and Knee Point Prediction Using Electrochemical Impedance Spectroscopy for Lithium Metal Battery Degradation via Machine Learning

**DOI:** 10.1002/advs.202502336

**Published:** 2025-05-05

**Authors:** Qianli Si, Shoichi Matsuda, Yasunobu Ando, Toshiyuki Momma, Yoshitaka Tateyama

**Affiliations:** ^1^ Department of Nanoscience and Nanoengineering Faculty of Science and Engineering Waseda University 3‐4‐1 Okubo Shinjuku‐ku 169‐8555 Japan; ^2^ Research Center for Energy and Environmental Materials (GREEN) National Institute for Materials Science (NIMS) 1‐1 Namiki Tsukuba Ibaraki 305‐0044 Japan; ^3^ NIMS‐SoftBank Advanced Technologies Development Center National Institute for Materials Science (NIMS) 1‐1 Namiki Tsukuba Ibaraki 305‐0044 Japan; ^4^ Laboratory for Chemistry and Life Science Institute of Integrated Research Institute of Science Tokyo 4259 Nagatsuta‐cho Midori‐ku Yokohama 226‐8501 Japan

**Keywords:** degradation, electrochemical impedance spectroscopy, knee points, lithium‐metal batteries, machine learning models

## Abstract

Lithium‐metal batteries (LMBs) are emerging as a promising next‐generation energy storage due to their exceptionally high energy density. However, accurately predicting their performance remains challenging because of the complex degradation mechanisms. In this study, a machine learning (ML) framework is proposed that combines electrochemical impedance spectroscopy (EIS) with the XGBoost algorithm to develop two predictive models: one for estimating capacity degradation and another for detecting the knee point (KP)—a critical inflection point in the degradation trajectory. SHapley Additive exPlanations (SHAP) analysis is employed to interpret feature importance, revealing that low‐frequency imaginary impedance components—associated with diffusion‐limited processes such as lithium depletion and accumulation—are most influential for capacity estimation. Conversely, high‐frequency features related to charge transfer resistance play a dominant role in the KP detection. To reduce data complexity and improve model efficiency, the input by selecting specific frequency points based on SHAP values is further optimized. The optimized models exhibit comparable or improved accuracy compared to those using the whole EIS data and have reasonable performance on unseen test data. The findings highlight that EIS‐based ML models can accurately forecast heaslth of LMBs, providing deeper insights into their aging processes and enhancing battery management strategies.

## Graphical abstract

This work explores a machine learning (ML) model with electrochemical impedance spectroscopy (EIS) data to address two critical challenges in Lithium metal batteries (LMBs): capacity degradation trajectory and knee point (KP) estimation, for the first time. By integrating data‐driven ML methods with physics‐based insights into EIS behavior, we provide a robust framework for LMB degradation prediction.

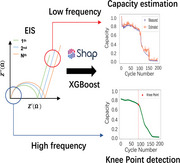

## 1 Introduction

Lithium‐ion batteries (LIBs) have become foundational in powering a wide range of modern technologies, from consumer electronics to electric vehicles (EVs) and energy storage systems.^[^ ^1^ ^]^ As renewable energy adoption continues to grow, there is an increasing demand for batteries with improved performance metrics, including higher energy density, enhanced safety, and longer cycle life. However, conventional LIB technology often faces significant difficulties, such as capacity degradation and increased impedance, particularly under high energy‐density designs or extended operational cycles.^[^ ^2^ ^]^ In critical applications, such as EVs, batteries typically need replacement once their capacity drops below 80%, making accurate predictions of battery state of health (SoH) and remaining useful life (RUL) essential to prevent unexpected failures and optimize battery use.^[^ ^3^ ^]^ The urgency for reliable, rapid battery health assessment is also driven by the need for effective recycling and replacement practices. To sustain this transformative energy shift, there is an urgent need to develop advanced battery chemistries and efficient health estimation technologies.

One promising alternative to LIBs is the lithium metal battery (LMB), which, with its lithium metal anode, offers a significantly higher theoretical specific capacity (3860 mAh g⁻¹ compared to 372 mAh g⁻¹ for graphite in LIBs) and a lower electrode potential (−3.04 V vs standard hydrogen electrode).^[^ ^4^ ^]^ The practical use of LMBs, however, is hindered by the limited chemical and mechanical stability of lithium metal. Additionally, most liquid electrolytes undergo decomposition on the lithium surface, resulting in an unstable solid electrolyte interphase (SEI) and the persistent consumption of lithium and electrolyte. These issues compromise battery safety and reduce cycle life. These instabilities are influenced by a wide range of factors, including cell chemistry, cell design parameters, and operating conditions. The complexity of these factors makes it challenging to efficiently explore and optimize battery performance.^[^ ^5^ ^]^

Traditional battery aging studies were based on modeling microscopic degradation processes, such as the growth of SEI,^[^ ^6^ ^]^ lithium plating,^[^ ^7^ ^]^ and the loss of active material.^[^ ^8^ ^]^ While these models provide valuable physical insights, it is impractical to characterize and simulate every degradation mechanism due to their complexity. To overcome this limitation, recent studies have shifted toward data‐driven approaches. Previous research has often focused on features extracted from cycle data,^[^ ^9^, ^10^ ^]^ yet data‐driven methods face challenges in selecting physically meaningful inputs and developing robust statistical models. Data‐driven studies on aging in LMBs remain limited, however, our previous study was able to predict the cycle life of LMBs using features derived from the cycle test data.^[^ ^11^ ^]^ Additionally, Liu et al. introduced a sequential explainable learning framework (SELF) to facilitate interpretable analysis of accelerated aging patterns in LMBs. Using data from just the first 10 cycles, SELF achieved a test error of 15.2% in predicting the knee point (KP) of the capacity degradation trajectory.^[^ ^12^ ^]^

In contrast to conventional current/voltage data, electrochemical impedance spectroscopy (EIS) provides rich insights into material properties, interfacial phenomena, and electrochemical reactions by measuring the current response to a voltage perturbation across a wide range of frequencies (including real part, imaginary part).^[^ ^13^, ^14^ ^]^ EIS effectively captures changes in the cathode, anode, electrolyte, solid electrolyte layer, and other components of LIBs throughout the aging process.^[^ ^15^ ^]^ Research combining EIS and data‐driven methods has been done in the past few years. For instance, Zhang et al. employed an extensive dataset of impedance spectra within a Gaussian process regression model to forecast battery capacity and estimate its lifespan.^[^ ^16^ ^]^ Similarly, T. K. Pradyumna et al. estimated the capacity of LIBs combining convolutional neural networks and EIS.^[^ ^17^ ^]^ In addition, based on the dataset of Zhang et al., Xia et al. estimated the SoH using partial EIS features.^[^ ^18^ ^]^

However, the existing research primarily focuses on well‐established, commercially available LIBs, where the capacity degradation follows relatively stable trends. In contrast, LMBs exhibit much more complex and nonlinear degradation behaviors due to the instability associated with the use of lithium metal as the anode. As such, LMBs face significant challenges, including dendrite growth and rapid capacity loss,^[^ ^19^ ^]^ particularly as the battery approaches the KP, a critical point in the degradation trajectory where accelerated aging begins.

To date, most existing approaches have been limited to stable, commercial LIBs,^[^ ^20^, ^21^, ^22^ ^]^ leaving a significant gap in the literature regarding the application of EIS for the performance prediction of LMBs. Furthermore, while some studies have focused on predicting the degradation trajectory or the remaining capacity for LIBs using EIS,^[^ ^16^, ^17^, ^18^, ^23^ ^]^ no work has yet been conducted to predict the KP. To fill this gap, in this study, we integrate EIS data, the Extreme Gradient Boosting (XGBoost) machine learning (ML) algorithm,^[^ ^24^ ^]^ and SHAP (SHapley Additive exPlanations)^[^ ^25^, ^26^ ^]^ to predict the discharge capacity and the KP of LMBs. The ML models are built using data from 16 LMBs, with 8 batteries used for training and seven out of the other eight for testing (excluding one due to its unusual degradation behavior). By carefully selecting specific EIS features, such as the low‐frequency range and the imaginary components for the capacity estimation, and high‐frequency components alongside the real impedance part for the KP prediction, we demonstrate that tailored feature selection can significantly enhance model accuracy, particularly when applied to the challenging and often unstable behavior of LMBs. By developing efficient and interpretable models, we aim to improve the accuracy of predictions, providing valuable insights into battery aging processes and advancing the development of predictive tools for more effective battery management and performance optimization.

## 2 Experimental Section

### 2.1 LMB Fabrication and Data Extraction

In our project, total 16 single‐layer stacked pouch‐type LMB cells (8 as training dataset, 8 as testing dataset) were assembled, consisting of a positive electrode made of LiNi_0.8_Mn_0.1_Co_0.1_O_2_  (40 mm × 30 mm) with mass loading of 30 mg cm^−2^, a separator (46 mm × 36 mm), and a negative electrode comprising a 50 µm thick layer of lithium on a 10 µm thick copper (Cu) current collector (42 mm × 32 mm). The details of cell components were described in our previous reports.^[^ ^25^, ^26^ ^]^ All cells were assembled in a dry room with a dew point below −50 °C, and electrolyte injection took place in an Ar‐filled glovebox with a dew point below −85 °C.

**Figure 1:**
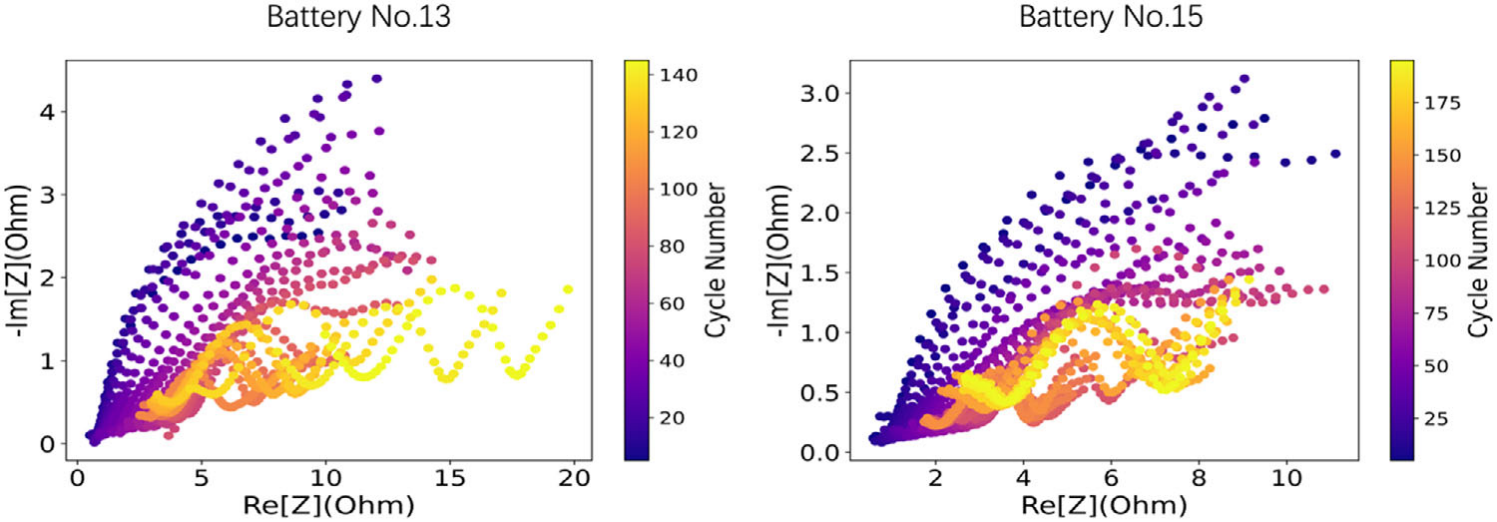
Impedance spectra for Battery No. 13 and Battery No. 15. The color bar indicates different cycles.

The fabricated cells were subjected to repeated charge/discharge cycle tests with different 8 kinds of conditions. For each condition, two cells were evaluated. The data for one cell was used as a training machine learning model, while the other was used to test the model. The details of the cycling test condition are summarized in Table S1 (Supporting Information). The charge/discharge cycle test of the cells was conducted with a constant current within a voltage range of 2–4.2 V, which basically underwent 100–200 cycles, and the capacity data were recorded continuously. The EIS test was conducted at 8 states which include 1) charge capacity 0 mAh (fully discharged state), 2) charge capacity 24 mAh, 3) charge capacity 48 mAh, 4) charge at 4.2 V (full charge voltage), 5) discharge capacity 0 mAh (fully charged state), 6) discharge capacity 24 mAh, 7) discharge capacity 48 mAh, and 8) discharge at 2 V (the cut‐off voltage of discharge). In each state, the frequency ranges were selected between 0.009 HZ and 10 KHZ with a total of 36 frequency points which are specified in Table S2 (Supporting Information). The corresponding EIS data at the frequency points are used as features in this project. Detailed information about these features can be found in the supplementary Excel file. However, during some of the states, the EIS data of some of the batteries were incomplete with respect to the cycles except for states: charge capacity 0 mAh, charge at 4.2 V, and discharge capacity 0 mAh. Hence, these three states are selected for further research. Here, we displayed the impedance spectra for batteries No. 13 and 15 at the fully discharged state in **Figure** **1**, the other batteries’ impedance spectra are shown in Figure S1 (Supporting Information). EIS spectra were plotted at regular intervals from cycles 5, 10, 15, 20, …, up to the final cycle, the *x*‐axis represents the real part, versus the negative imaginary part in *y*‐axis. Each battery shows distinct changes in impedance spectra as they age. The raw experimental EIS data are then utilized as features in the current work.

### 2.2 Machine Learning Process

Totally, two ML models were constructed in this work, EIS‐Capacity model and EIS‐KP model. The EIS‐Capacity model is a ML framework designed to estimate LMB capacity using the EIS data. The training dataset comprises EIS samples aggregated from all training batteries, structured as a matrix with dimensions 1050 × 72. Here, each row represents a single battery cycle, and the 72 input features correspond to the impedance spectra components: the first 36 columns are the real parts, while the imaginary parts are listed in the remaining 36 columns. The target values are the measured capacities of the training batteries, establishing a direct mapping between impedance characteristics and capacity degradation. For model evaluation, the measured capacities of the test battery serve as ground truth, which are compared with the model's predicted capacities to assess generalization performance. This framework ensures that the model learns the relationship between impedance features and capacity evolution across cycles, enabling robust state‐of‐health estimation.

The EIS‐KP model aims to predict the KP, a critical transition in capacity degradation rate, using the EIS data. The training dataset consists of 572 EIS samples (572 × 72 matrix), where each sample corresponds to a cycle preceding the KP detection in the training batteries. The target values are the remaining cycle numbers until the KP occurs, derived from the Kneed algorithm. This algorithm identifies the KP as the point of maximum curvature in the capacity degradation curve, ensuring an objective and reproducible reference. During testing, the model predicts KPs for test batteries based solely on their EIS data. The actual KPs for testing batteries are independently determined using the Kneed algorithm, serving exclusively as a validation benchmark. This approach ensures that predictions are based on learned impedance‐degradation relationships, while predicted KP provides an unbiased evaluation of model accuracy.

To develop both models, XGBoost regressor was employed, a widely recognized machine learning algorithm known for its efficiency, scalability, and predictive accuracy. XGBoost constructs an ensemble of decision trees sequentially, where each successive tree corrects the residual errors of its predecessors, optimizing the model by minimizing a specified loss function. The algorithm incorporates several advanced techniques, including regularization, tree pruning, and parallelization, to enhance performance and mitigate overfitting. Due to its speed, robustness, and ability to handle large, complex datasets, XGBoost is extensively used in both research and industrial applications. Moreover, XGBoost provides valuable insights into feature importance, allowing for the identification of key EIS features that contribute most significantly to the model's predictions. To ensure optimal performance, a grid search with 5‐fold cross‐validation was conducted for hyperparameter tuning. A detailed explanation of the XGBoost algorithm and parameter optimization are provided in Notes S1 and S2 (Supporting Information) respectively in the Supporting information.

To evaluate the performance of the machine learning models, the following three performance indices were implemented.

1. Mean absolute error (MAE)

(1) $$MAE=\frac{1}{n}\sum_{i=1}^{n}\left|y_{i}-{\hat{y}}_{i}\right|$$

2. Root mean square (RMSE)

(2) $$RMSE=\sqrt{\frac{1}{n}\sum_{i=1}^{n}{\left(y_{i}-{\hat{y}}_{i}\right)}^{2}\;\;}$$

3. Coefficient of determination (R^2^)

(3) $$R^{2}=1-\frac{\sum_{i=1}^{n}{\left(y_{i}-{\hat{y}}_{i}\right)}^{2}}{\sum_{i=1}^{n}{\left(y_{i}-\bar{y}\right)}^{2}}$$

here, n represents the number of samples, *y_i_* and ${\hat{y}}_{i}$ represents the actual values and the predicted values. The MAE measures how close the predictions are to the actual outcomes. In contrast, the RMSE captures the spread of errors and is more sensitive to larger discrepancies compared to MAE. Smaller values for both MAE and RMSE indicate better model performance, while higher values suggest poorer predictions. The coefficient of determination R^2^ metric, expressed as a percentage, ideally reaches 100% or 1, indicating a strong correlation between the observed and predicted values.

### 2.3 XGBoost Feature Importance (XFI) Analysis

XFI analysis is a powerful method for identifying the key features in a dataset, which have the greatest influence on the model's predictions. By understanding which features are most important, researchers can enhance model performance, reduce complexity, and gain valuable insights into the underlying variables driving the predictions. XGBoost provides several methods to calculate feature importance scores, each offering a unique perspective on feature contributions. One such method is the weight approach, which evaluates how often each feature is used to split nodes across all decision trees in the model. Features that are utilized more frequently during the splitting process are considered more important, as they play a more significant role in the model's decision‐making. This method highlights the features that the model relies on most heavily during training.

In this project, we visualize the XGBoost Feature Importance (XFI) scores using a heatmap. These scores are intrinsic to the XGBoost model and reflect the relative contribution of each feature to the model's predictions. The heatmap is generated by computing saliency values, which are derived by averaging the XFI scores across all test samples. This provides a measure of the average importance of each feature, enabling a robust and interpretable identification of the most influential features. The heatmap is structured such that the x‐axis represents the cycle number of the battery, the y‐axis represents the features, and the color intensity corresponds to the importance of each feature. This visualization not only facilitates the interpretation of the relative significance of features across different test samples but also reveals patterns in feature contributions. By analyzing these patterns, we gain deeper insights into the relationships between features and the target variable, enhancing our understanding of the factors driving the model's predictions.

### 2.4 SHAP Feature Importance (SFI) Analysis

SHAP is a game‐theory‐based approach that provides a unified and interpretable measure of feature importance by quantifying the contribution of each feature to the model's predictions. Unlike traditional feature importance methods, which typically rank features based on their average impact, SHAP offers a more granular and detailed explanation of how each feature influences individual predictions. This capability makes SHAP particularly valuable for understanding complex, nonlinear relationships in ML models, as it not only identifies important features but also explains their contribution to specific predictions.

In this work, SFI analysis was applied to both the capacity estimation model and the KP prediction model to evaluate the relative contribution of each EIS feature. Specifically, SFI scores were computed to identify the most influential EIS features affecting capacity degradation and KP prediction. Unlike traditional feature importance methods, which only rank features based on their average impact, SFI offers a more detailed and interpretable explanation of each feature's influence on individual predictions. This method allows us to visualize the distribution of feature importance across different battery cycles, providing deeper insights into which features play the most significant role in predicting battery behavior.

**Figure 2:**
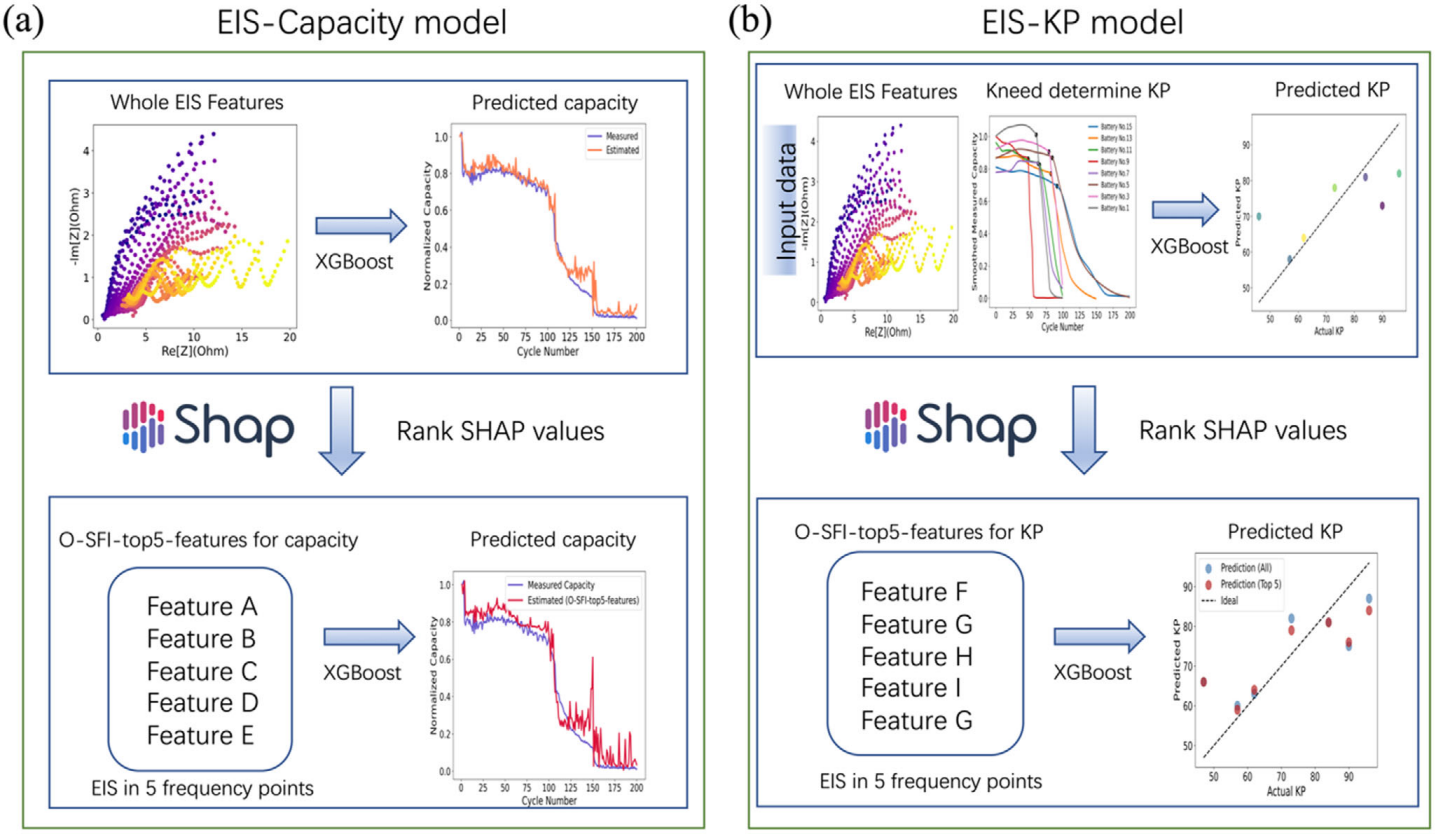
Schematic flow of a) EIS‐Capacity model and b) EIS‐KP model.

**Figure** **2** illustrates the schematic workflows of these two models. Initially, the whole EIS dataset was integrated with the XGBoost ML algorithm to predict capacity and KP. Subsequently, SHAP analysis was employed to identify and extract key features contributing to model performance. Using these selected features, simplified machine learning models were constructed, ensuring interpretability while maintaining predictive accuracy. The workflows highlight the systematic approach taken to address the challenges of LMB degradation prediction.

## 3 Results and Discussion

### 3.1 LMB Capacity Estimation by EIS

**Figure 3:**
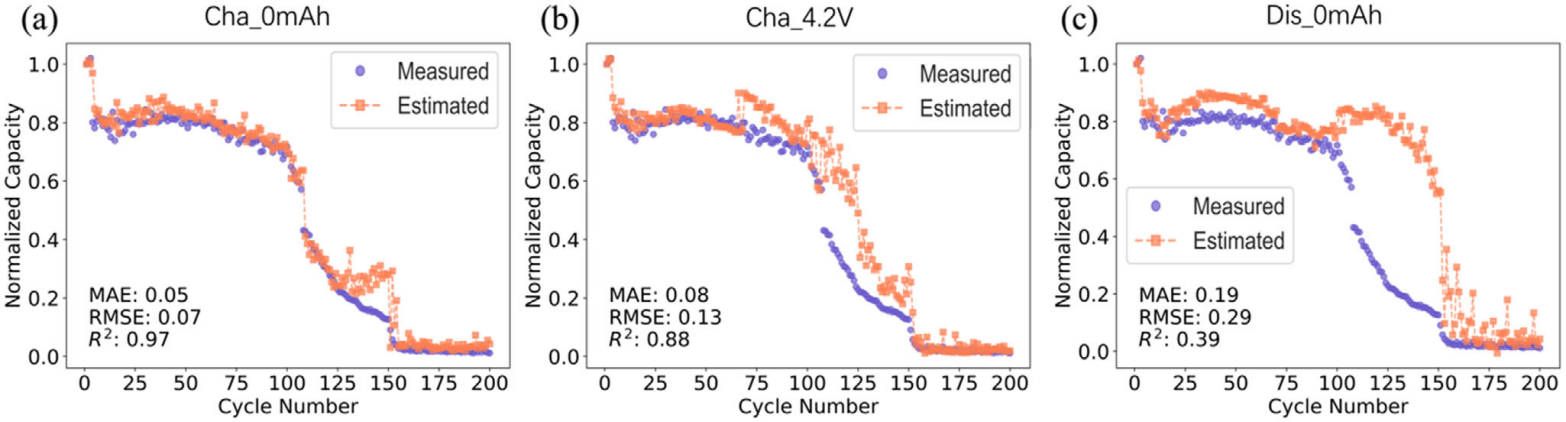
Estimated (orange) and Measured (purple) capacity as a function of cycle number for battery No. 16 in three different states.

In this part, we conducted the capacity estimation of the LMBs using EIS from different states. As we illustrated before, a charge capacity 0 mAh, a charge at 4.2 V, and a discharge capacity 0 mAh states are chosen to estimate the capacity separately. The EIS of batteries No. 1, 3, 5, 7, 9, 11, 13, and 15 in different states were selected to train the model and the rest of the 8 batteries No. 2, 4, 6, 8, 10, 12, 14, and 16 were used to test the model respectively. The criterion for classification of the training set from the test set is that the charge and discharge current densities of the batteries in both sets are identical respectively (for example, battery 1 and battery 2 have the same discharge and charge densities which is also shown in the supporting information). In **Figure** **3**, it displays the prediction results for battery No. 16 using the three different states.

The figures indicate that predictions based on EIS data from the Cha_0mAh state outperform those from the other two states, exhibiting the highest R^2^ and the lowest MAE and RMSE values. Parity plots for the predictions of the remaining seven batteries using Cha_0 mAh state are provided in Figure S2 (Supporting Information). **Table** **1** summarizes the R^2^ values for different batteries in various states. Notably, the R^2^ values for cell 2 are outside the expected range of 0–1, appearing as negative values, which reflect very poor predictive performance. Upon analysis, we observed that, despite battery 1 and battery 2 sharing the same charge and discharge current densities, their capacity degradation trajectories differ significantly, resulting in poor prediction accuracy for cell 2. Therefore, the capacity estimation of Battery 2 in this work is excluded, we focus on the remaining seven cells. The R^2^ values for the remaining seven batteries are presented in Table 1. Overall, the Cha_0mAh state provides the best estimation performance. Therefore, the following research will focus specifically on this state.

**Table 1:** Summary of the R^2^ of the testing batteries under 3 different states.

Cell	R^2^: Cha_0mAh	R^2^: Cha_4.2V	R^2^: Dis_0mAh
2	−106	−110	−117
4	0.971	0.974	0.976
6	0.912	0.958	0.973
8	0.799	0.772	0.751
10	0.936	0.994	0.992
12	0.900	0.784	0.835
14	0.971	0.920	0.965
16	0.969	0.884	0.392
Average	0.923	0.898	0.841

**Figure 4:**
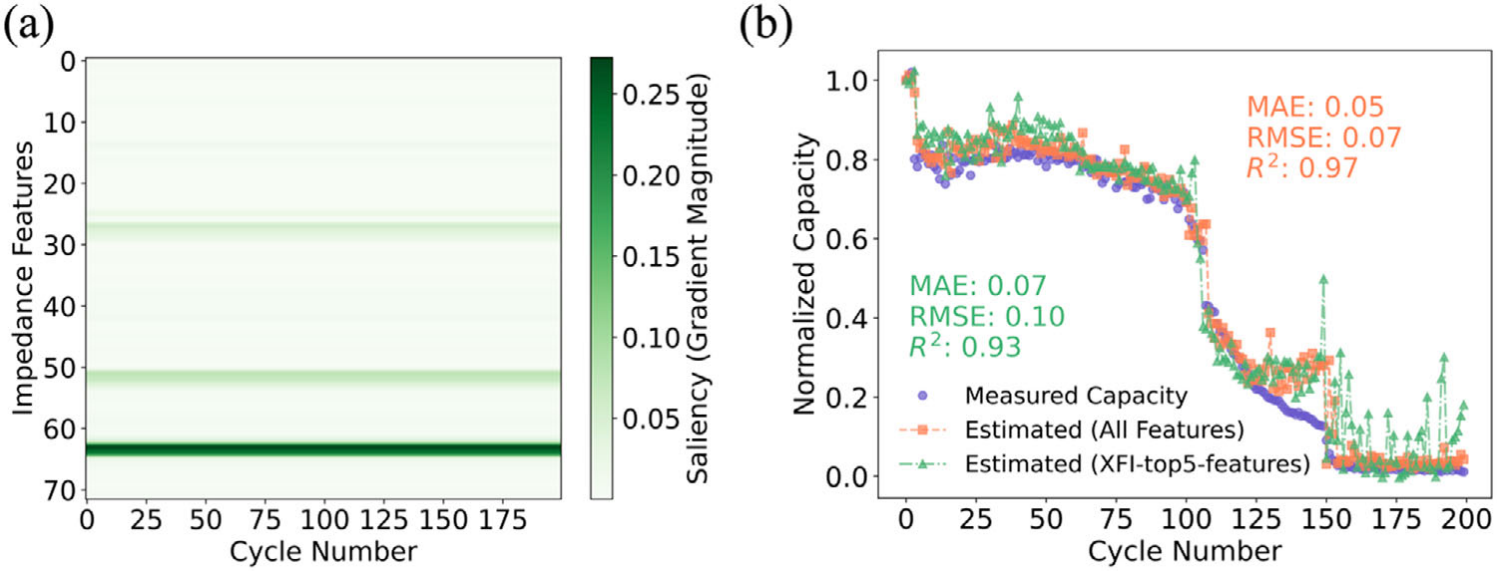
a). In the XFI heat map of Battery No. 16, the *x*‐axis indicates the cycle numbers of the battery, the *y*‐axis represents the impedance data at 36 frequency points ranging from 0.0093 HZ to 10 KHZ (bottom to top). b). Comparison of the estimation results of battery 16 using the whole EIS data and the top five most important EIS features.

The capacity estimation model is developed using the XGBoost ML algorithm, one of the key strengths is its ability to evaluate feature importance, making it especially valuable for applications where understanding the contribution of each input feature to the model's predictions is critical. Using the XFI analysis introduced in Section 2.3, we assess the relative importance of EIS features. This approach enhances interpretability by highlighting the features that contribute most significantly to capacity estimation. **Figure** **4**a shows the XFI heat map for Battery No. 16. Evaluating this map provides a direct visualization of how the most informative frequency points are associated with capacity estimation. Four of the top five most important features (XFI‐top5‐features) are concentrated at low‐frequency points (specifically the 63rd, 64th, 51st, and 52nd), while the 28th feature stands out as being located at a relatively high‐frequency point (refer to the supplementary Excel file for detailed numbering), which may provide unexpected special correlation. Then we utilized XFI‐top5‐features as the input features to estimate the capacity of the testing batteries. The estimation results of battery No.16 are shown in the Figure 4b, for the other batteries, the estimation results are shown in Figure S3 (Supporting Information).

Through the analysis of Figure 4b, we observe that the estimation accuracy using the XFI‐top5‐features shows a slight decrease compared to the full EIS dataset. The observed reduction in R^2^ when utilizing only the top features reflects a well‐known trade‐off between model complexity and generalizability. While the model trained with the complete feature set achieves a higher R^2^, suggesting superior performance on the current dataset, it may also be more prone to overfitting due to its dependence on a larger number of features. In contrast, the model using the XFI‐top5‐features exhibits a modest reduction in performance but may demonstrate better generalization by concentrating on the most impactful features. Interestingly, the predictive accuracy improved when using the reduced feature set for certain test batteries, such as Battery No. 4 and Battery No. 6. This underscores the complexity and variability of XFI analysis across different samples, suggesting that the optimal feature set can vary depending on the specific characteristics of the testing batteries.

Furthermore, for all other test batteries, the XFI‐top5‐features were consistent with those of Battery No. 16. This consistency highlights a limitation of the XFI analysis, which provides a global ranking of feature importance but lacks the ability to offer detailed, sample‐specific insights into how individual features influence predictions. Despite these limitations, our findings demonstrate that capacity estimation may be achieved using a subset of carefully selected frequency points from the EIS data. This insight suggests that focusing on specific frequency regions, rather than the entire EIS spectrum, could be an effective strategy for improving prediction efficiency while maintaining accuracy. Hence, finding a sample‐specific explanation of how each feature influences the model's prediction for individual test samples is important.

**Figure 5:**
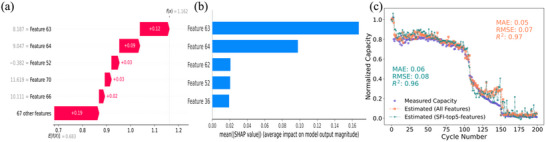
a). Waterfall plot illustrating the contribution of each impedance feature, ranked by SHAP value, to the first‐cycle capacity estimation of battery No. 16. b). Ranking of impedance features based on their importance across the battery's entire life cycle by the order of SHAP values. c). Comparison of the estimation results for battery 16 using the whole EIS features versus SFI‐top5‐features identified by SHAP values.

In this study, the SHAP method was employed to evaluate and rank the contributions of 72 impedance features to capacity estimation for various test batteries. **Figure** **5**a illustrates the contribution of each impedance feature to the first‐cycle capacity estimation. The estimated capacity value, f(x) = 1.162, is displayed in the top‐right corner, while the gray numbers on the left indicate the SHAP values of the features, ranked from highest to lowest. Notably, the top five ranked features account for a significantly larger contribution compared to the cumulative contributions of the remaining 67 features.

Figure 5b presents the mean absolute SHAP values of SFI‐selected top five ranked features (SFI‐top5‐features) for Battery No. 16 across all cycles of capacity estimation. Based on this analysis, the impedance features 63, 64, 62, 52, and 36 are identified as the most influential for Battery No. 16. For other test batteries, SFI‐top5‐features differ, as shown in Table S3 (Supporting Information). This variability reflects the battery‐specific nature of feature importance as determined by SFI. The identified SFI‐top5‐features for each battery were subsequently used as inputs to the ML model for re‐evaluation of capacity estimation.

Figure 5c compares the capacity estimation results for Battery No. 16 using the SFI‐top5‐features versus using the whole EIS features. The results indicate that for Battery No. 16, capacity estimation using whole EIS features outperformed the SFI‐top5‐features. The parity plots of other batteries are shown in Figure S4 (Supporting Information). A summary of the capacity estimation for all test batteries, including comparisons between the SFI‐top5‐features, XFI‐top5‐features, and whole EIS features is provided in **Table** **2**.

**Table 2:** Summary of the R^2^ of the testing batteries using whole EIS features, XFI‐top5‐features, and SFI‐top5‐features.

Cell	R^2^: Whole EIS	R^2^: XFI‐top5‐features	R^2^: SFI‐top5‐features
4	0.971	0.978	0.960
6	0.912	0.921	0.942
8	0.799	0.695	0.743
10	0.936	0.896	0.926
12	0.900	0.902	0.869
14	0.971	0.924	0.962
16	0.969	0.932	0.960
Average	0.914	0.893	0.908

From the results in Table 2, it can be observed that the capacity estimation for Battery No. 6 and Battery No. 8 shows improvement when using SFI‐top5‐features compared to both the whole EIS features and XFI‐top5‐features. For Battery No. 10, No. 12, and No. 14, the estimation results with SFI‐top5‐features es are slightly improved or comparable but remain less accurate than those obtained using the whole EIS features. Overall, the SFI‐top5‐features demonstrate superior performance compared to the XFI‐top5‐features. Further analysis reveals that although the SFI‐top5‐features differ across the test batteries, they are consistently located within the low‐frequency region of the impedance spectrum. Additionally, all these features are derived from the imaginary component of the impedance data. An exception is Battery No. 10, where one of the SFI‐top5‐features (the 28th feature) is extracted from the real component of the impedance data.

**Figure 6:**
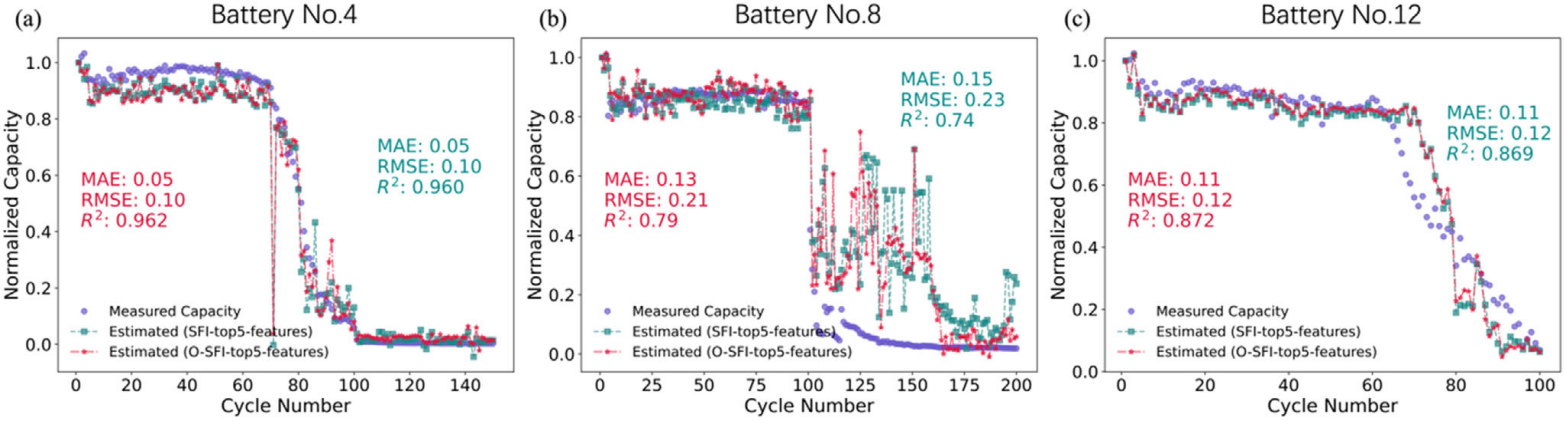
Comparison of the estimation results using SFI‐top5‐features and O‐SFI‐top5‐features on three different Batteries a) Battery No. 4, b) Battery No. 8, and c) Battery No. 12.

Finally, to simplify the model while simultaneously enhancing its generalizability, we aggregate the SFI‐top5‐features identified for each test battery. By summing the SHAP values of the SFI‐top5‐features across all testing batteries, we identify the top 5 features with the highest “overall” SHAP values (O‐SFI‐top5‐features), representing those with the greatest impact across the entire dataset, which is detailed in the Table S4 (Supporting Information). The O‐SFI‐top5‐features are 63, 64, 52, 51, and 53 subsequently, which are all extracted from the low‐frequency region and imaginary part of the EIS data, the corresponding frequency points are 0.2352, 0.1583, 18.08, 8.121, and 12.18 Hz respectively. These O‐SFI‐top5‐features are utilized to build the LMB capacity estimation model. **Figure** **6**, displays the estimation results of using the O‐SFI‐top5‐features of three testing batteries, for the other 4 batteries, the results are shown in Figure S5 (Supporting Information).

Through the analysis, it was observed that the EIS‐Capacity model employing the O‐SFI‐top5‐features generally yielded superior or comparable performance compared to the model utilizing the SFI‐top5‐features. However, slight decreases in predictive accuracy were observed for Battery No. 10, No. 14, and No. 16

For these three batteries, the discrepancy can be attributed to several factors. In the case of Battery No. 10, which was subjected to a high charging rate of 3.0 mA cm⁻^2^ and a low discharging rate of 0.6 mA cm⁻^2^, this charge/discharge protocol has been demonstrated to accelerate battery degradation significantly in previous research. As illustrated in Figure S4b, its measured capacity degradation trajectory exhibits significant fluctuations between cycles 50 and 100—an anomaly not observed in the degradation patterns of other test batteries. This suggests that Battery No. 10 follows a distinct degradation behavior. Additionally, since the O‐SFI‐top5‐features were selected based on the overall battery population, they may not fully capture the unique degradation characteristics of individual batteries. Conversely, the SFI‐top5 features include specific variables (e.g., Feature 28 for Battery No. 10) that are particularly relevant for capturing the irregular pattern, thereby enhancing predictive accuracy. Feature 28, in particular, likely plays a crucial role in representing the fluctuations observed in Battery No. 10′s degradation trajectory. In addition, in the previous research,^[^ ^27^ ^]^ the high charge current density, and low discharge current density will make the battery degrade severely as proved by experiment.

Similarly, for Battery No. 14 and Battery No. 16, features 51 and 53 are not among their respective SFI‐top5‐features, which may explain the reduced prediction performance when using the O‐SFI‐top5 ‐features. Despite this, the overall performance remains reasonable. The primary objective of this study is to reduce the number of input features while maintaining reliable predictive accuracy. The results indicate that, although there are some exceptions, the O‐SFI‐top5 features still provide satisfactory performance across most cases, demonstrating the feasibility of feature reduction without significantly compromising predictive capability.

This finding supports the hypothesis that focusing on specific segments of the EIS data, particularly the low‐frequency region and the imaginary component, is sufficient to achieve high capacity estimation accuracy. This approach not only simplifies the model by reducing the number of input features but also enhances its generalizability, providing a more efficient and interpretable framework for capacity estimation in LMBs.

### 3.2 LMB Knee Point Estimation by EIS

LMBs examined in this study demonstrate distinct nonlinear capacity degradation, presenting unique challenges for accurate lifetime prediction and effective battery management, unlike the commercialized lithium‐ion counterparts. This nonlinear degradation is characterized by an initial phase of gradual capacity decline, followed by a critical transition into a region of rapid capacity loss. The transition point, commonly referred to as KP, is crucial as it marks the onset of accelerated aging and reduced performance. While notable advancements have been made in predicting the KP for lithium‐ion batteries, this task is particularly complex for LMBs due to their less mature state of research and more intricate degradation behaviors.

In this study, we developed a ML model to predict the KP for LMBs using the EIS data, the KP prediction model follows the same framework as the capacity estimation model, utilizing EIS data recorded at the charge capacity 0 mAh. Given the substantial fluctuations observed in the degradation curves of our batteries, we employed the Locally Weighted Scatterplot Smoothing (LOWESS) algorithm to enhance the clarity of the capacity degradation trends which has been used in the previous work. ^[^ ^28^ ^]^ This smoothing technique was applied to normalize the measured capacity data and estimated capacity across the cycle range, using a smoothing parameter set to 0.2. This parameter determines the proportion of data points included in each localized regression, striking a balance between preserving local variations and generating a general trend. The smoothed capacity data was then utilized for further analysis and visualization, effectively reducing noise and highlighting the underlying degradation patterns.

**Figure 7:**
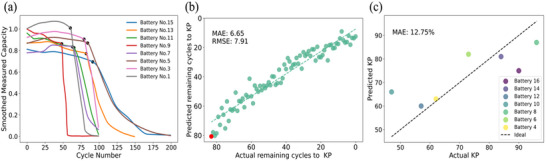
a) Smoothed measured capacity degradation trajectories and corresponding KP (black dots) for the training batteries. b) Actual versus predicted remaining cycles to KP for Battery No. 14. c) Actual versus predicted KPs for all testing batteries.

**Figure** **7**a illustrates the smoothed measured capacity degradation trajectories and their corresponding KPs for the training batteries. Figure 7b presents the prediction results of battery No. 14 obtained solely from the EIS data, excluding capacity variation considerations. Here, the x‐axis represents the actual KP, plotted in reverse to reflect a countdown from the traditional perspective, with the y‐axis following the same reversed orientation. The data points in this figure indicate the remaining cycles until the KP occurs. However, we primarily focus on the prediction result for the first cycle, as this represents the mathematically precise point at which the KP occurs in the degradation trajectory, which is highlighted in red. Figure 7c illustrates the comparison between the actual and predicted KP for all testing batteries. The mean absolute error (MAE) is 12.75%, demonstrating improved accuracy to the prior work by Liu et al.,^[^ ^12^ ^]^ which predicted LMB KP using discharge voltage curves.

**Figure 8:**
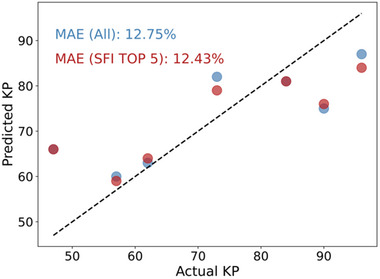
Comparison of prediction results using different input feature sets. The blue dots represent the results obtained using the whole EIS feature set, while the red dots indicate the outcomes based on the SFI‐top5‐features.

Here, we utilized SFI analysis to analyze the impact of feature selection on model performance for KP prediction. SFI analysis revealed that, across different testing batteries, SFI‐top5‐features remained consistent, specifically features 1, 0, 4, 37, and 56. This contrasts with the capacity estimation model, where the top features varied among batteries. Hence, we utilized the SFI‐top5 features from 10 kHz, 6.74 kHz, 2.063 kHz, 3.728 Hz to reconstruct the model. The comparison results, illustrated in **Figure** **8**, show that the MAE for the model using the SFI‐top5‐features is 12.43%, which is slightly lower than that of the model utilizing the entire EIS dataset and prior work. Given the inherent complexity of lithium‐metal battery degradation, our findings indicate that concentrating on specific EIS data regions, particularly high‐frequency components (except feature 56), is possible to achieve a relatively high level of accuracy in KP prediction.

Here, we notice for these two ML models, the contrasting feature selections for KP prediction and capacity estimation can be attributed to the distinct degradation processes influencing each task. KP prediction is primarily driven by high‐frequency components of the impedance (Feature 0, 1, 4, and 37), which capture rapid, early‐stage degradation mechanisms associated with the onset of accelerated capacity loss. In contrast, capacity estimation relies more on low‐frequency components and the imaginary part, which reflects long‐term degradation trends and overall battery health. These differences highlight the importance of selecting appropriate features based on the degradation phase being studied, with high‐frequency features being more relevant for predicting KP and low‐frequency features for estimating capacity. By focusing on the most relevant EIS segments for each task, both models achieve high accuracy while improving efficiency and generalizability.

### 3.3 Machine Learning Model Generalizability Validation

**Figure 9:**
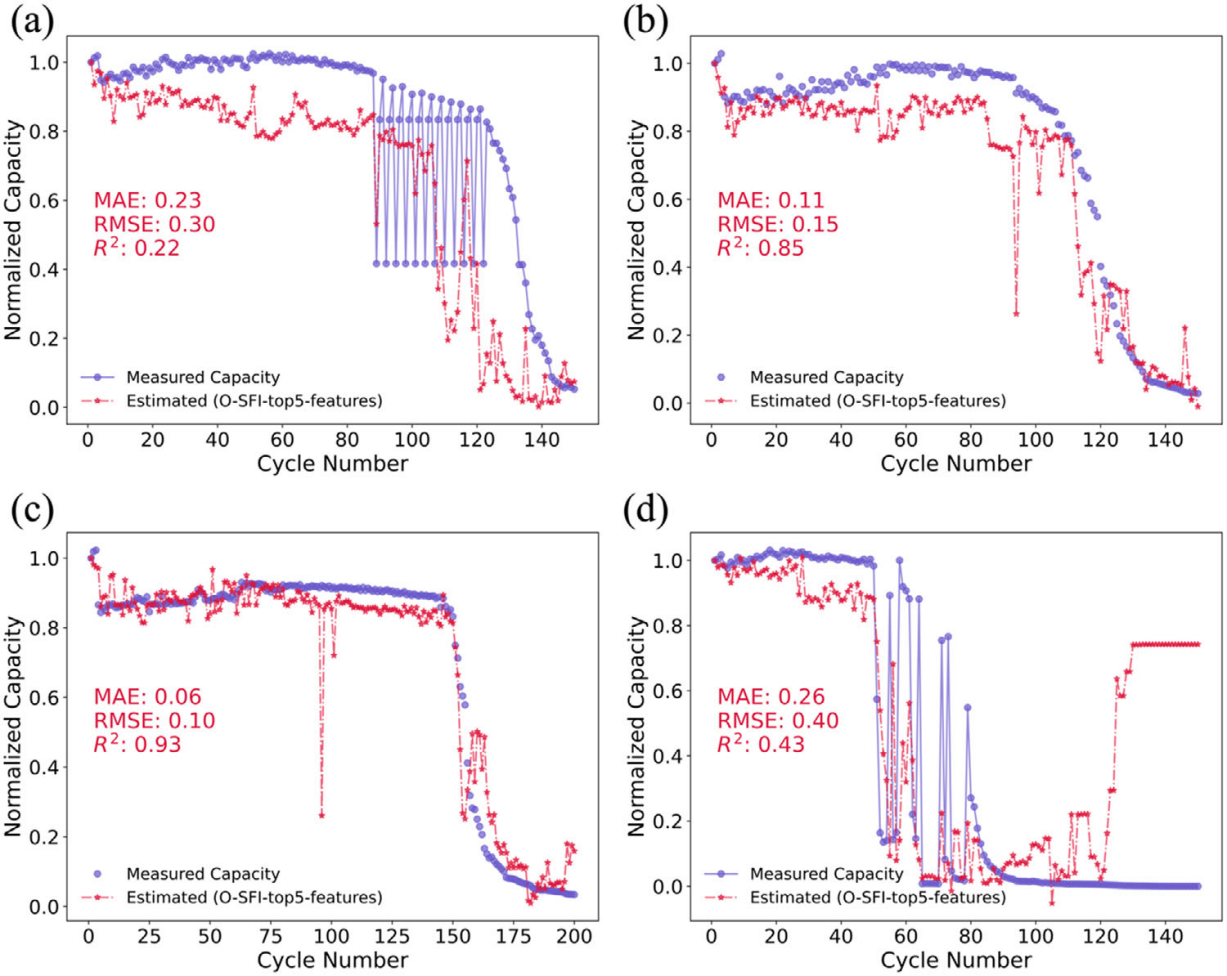
Comparison of the estimation results using O‐SFI‐top5‐features and Measured capacity on four unseen Batteries a) Battery A, b) Battery B, c) Battery C, and d) Battery D.

After we build the ML model using the partial EIS features, we need to validate their generalizability. Here, we implemented our EIS‐Capacity and EIS‐KP models on 4 unseen batteries. These unseen batteries have different cycling test conditions compared to the previously used batteries in our ML models. The cycling test condition are shown in Table S1. In **Figure** **9**, it displays the prediction results of the unseen batteries using the EIS‐Capacity model.

The EIS‐Capacity model demonstrates strong predictive performance for Batteries B and C, mainly because their capacity degradation is gradual and similar to the patterns in the training data. They exhibit a relatively steady decline in capacity without abrupt drops or recoveries, resembling the degradation trajectories that the model has learned. In contrast, Batteries A and D show irregular degradation with sharp fluctuations—Battery A between cycles 90–125 and Battery D between 50 and 80. These anomalies are absent from the training dataset, which makes it difficult to accurately capture such unpredictable behaviors, resulting in lower prediction accuracy. The unusual degradation patterns are likely influenced by different discharge/charge rate (C‐rate) conditions, which differ considerably from those of the training batteries. Interestingly, Battery No. 10 also has strong fluctuations but is predicted more accurately because the training data includes Battery No. 9, which has a similar pattern. This highlights the value of including a wide variety of degradation behaviors in the training data.

The EIS‐KP model shows varied prediction accuracy across different test batteries, depending on their degradation patterns and similarity to the training data. For Batteries A and B, the model predicts KPs reasonably well, with small errors (e.g., Battery A: predicted 79 versus actual 84; Battery B: predicted 86 versus actual 95), resulting in an average MAE of 7.71%. Although Battery A's degradation pattern differs from those in the training dataset, its fluctuations are relatively regular, enabling the LOWESS smoothing method to retain critical inflection points and allowing the model to capture its actual KP with reasonable accuracy. In contrast, Battery D's KP is overpredicted (predicted: 80 versus actual: 50), likely due to artifacts introduced by the LOWESS smoothing method. Unlike Battery A's regular fluctuations, Battery D's degradation trajectory exhibits severe and irregular fluctuations. While smoothing mitigates noise in degradation trajectories, aggressive application to highly fluctuating data can obscure critical inflection points, particularly in batteries with rapid early degradation followed by stabilization. Battery C's KP is severely underestimated (predicted: 81 versus actual: 146), primarily because the training dataset lacks examples of batteries with KPs exceeding 100 cycles. Machine learning models inherently struggle to extrapolate beyond the scope of their training data, particularly for nonlinear degradation behaviors, leading to systematic underestimation in such cases.

These findings highlight the model's sensitivity to degradation patterns and the importance of comprehensive training data coverage. To enhance robustness, future research should prioritize expanding the diversity of the training dataset, incorporating batteries exhibiting a wider spectrum of degradation behaviors, cycle life ranges, and a wider range of C‐rate conditions, refining smoothing techniques to better capture key degradation features, and integrating physics‐informed constraints to improve extrapolation capability. Additionally, from a data quality perspective, the presence of batteries with severe and irregular fluctuations, such as Battery A and Battery D, is undesirable for both model training and evaluation, as these cases introduce inconsistencies that hinder the model's ability to learn meaningful degradation patterns. While Battery A's fluctuations are relatively regular and do not significantly impact prediction accuracy, Battery D's abrupt variations create substantial challenges in KP estimation. Such batteries with extreme fluctuations should ideally be excluded from both the training dataset and unseen test data to ensure a more reliable and generalizable model. Future studies should establish strict criteria for identifying and filtering out these outliers, focusing on training the model with batteries exhibiting physically meaningful degradation behaviors and incorporating diverse cathode materials in LMB to enhance the generalizability and robustness of the findings. This approach would allow the model to generalize effectively while maintaining robustness across real‐world applications.

## 4 Conclusion

In conclusion, this study presents a comprehensive investigation of the degradation behaviors of LMBs through ML models implementing EIS data. By addressing capacity estimation and KP prediction, we provide new insights into the electrochemical and physical processes governing LMB degradation.

For capacity estimation, the results demonstrate that low‐frequency impedance features, particularly those associated with the imaginary component, are critical for capturing the long‐term degradation behavior of LMBs. The dominance of low‐frequency features highlights their sensitivity to interfacial resistance and charge transfer processes, which are directly influenced by the lithium depletion/dendrite growth and electrolyte decomposition. These processes collectively dictate the gradual capacity decline observed during cycling. By identifying and focusing on these specific impedance regions, our model simplifies the feature set while maintaining high accuracy, underscoring the importance of the low‐frequency regime in reflecting the battery's health state.

In contrast, the prediction of KP—a crucial transition where capacity degradation accelerates—relies predominantly on high‐frequency features and the real component of the impedance. These features indicate the system's resistance and electronic conductivity changes, which are often linked to the early stages of SEI instability, pore‐clogging, and the onset of lithium plating. The observed shift in key features for KP prediction emphasizes the distinction between the early detection of rapid degradation mechanisms and the comprehensive assessment of long‐term capacity loss. Our findings demonstrate that high‐frequency impedance features are particularly effective in capturing the subtle structural and kinetic changes preceding KP, providing a valuable tool for proactive battery management.

A key finding of this study is the ability to minimize the required frequency range of the impedance spectra for LMB capacity and KP estimation, thereby significantly reducing measurement time. Unlike conventional approaches that rely on extracting features from the entire impedance spectrum, our method focuses on directly selecting the most relevant frequency range for measurement. As a result, while traditional techniques require capturing the full impedance spectrum, our approach streamlines the process by concentrating only on the essential frequency components. This enables the identification of the most informative impedance frequency points for battery health assessment, enhancing both the efficiency and accuracy of predictions. Our method offers significant potential for optimizing the use of EIS in real‐world LMB health monitoring.

In conclusion, this study enhances the understanding of LMB degradation by establishing connections between critical EIS features and specific physical and chemical degradation mechanisms. While there is room for further improvement in model robustness, these findings contribute to the improvements in measurement efficiency and prediction accuracy, paving the way for practical applications in advanced LMB prognostics and management

## Conflict of Interest

The authors declare no conflict of interest.

## Supporting information



Supporting Information

Supporting Information

## Data Availability

The data that support the findings of this study are available from the corresponding author upon reasonable request.
