# Supplementary material for: Capacity Estimation and Knee Point Prediction Using Electrochemical Impedance Spectroscopy for Lithium Metal Battery Degradation via Machine Learning
Source: Adv Sci (Weinh). 2025 May 5;12(27):2502336. doi: 10.1002/advs.202502336 (PMC12463089; doi:10.1002/advs.202502336)
Supplement: Supplementary file 1 — Supporting Information [file ADVS-12-2502336-s002.pdf]

ADVANCED SCIENCE
Open Access

Supporting Information

for *Adv. Sci.*, DOI 10.1002/advs.202502336

Capacity Estimation and Knee Point Prediction Using Electrochemical Impedance Spectroscopy for Lithium Metal Battery Degradation via Machine Learning

Qianli Si, Shoichi Matsuda, Yasunobu Ando, Toshiyuki Momma and Yoshitaka Tateyama**

Supporting Information

Capacity Estimation and Knee Point Prediction Using Electrochemical Impedance Spectroscopy for Lithium Metal Battery Degradation via Machine Learning

Qianli Si[a,b,*], *Shoichi Matsuda*[b,c], *Yasunobu Ando*[d], *Toshiyuki Momma*[a], *Yoshitaka Tateyama*[a,b,c,d,*]

a. *Department of Nanoscience and Nanoengineering, Faculty of Science and Engineering, Waseda University, 3-4-1 Okubo, Shinjuku-ku 169-8555, Japan*
b. *Research Center for Energy and Environmental Materials (GREEN), National Institute for Materials Science (NIMS), 1-1 Namiki, Tsukuba, Ibaraki 305-0044, Japan*
c. *NIMS-SoftBank Advanced Technologies Development Center, National Institute for Materials Science (NIMS), 1-1 Namiki, Tsukuba, Ibaraki 305-0044, Japan*
d. *Laboratory for Chemistry and Life Science, Institute of Integrated Research, Institute of Science Tokyo, 4259 Nagatsuta-cho, Midori-ku, Yokohama 226-8501, Japan*

Supplementary Table S1. Charge/discharge cycle test protocol used in this project

Battery ID	Cathode	electrolyte	Charge current density	Discharge current density
No. 1	NMC811	4M_LiFSI in DME	0.6	0.6
No. 2	NMC811	4M_LiFSI in DME	0.6	0.6
No. 3	NMC811	4M_LiFSI in DME	1.5	1.5
No. 4	NMC811	4M_LiFSI in DME	1.5	1.5
No. 5	NMC811	4M_LiFSI in DME	1.5	3
No. 6	NMC811	4M_LiFSI in DME	1.5	3
No. 7	NMC811	4M_LiFSI in DME	1.5	6
No. 8	NMC811	4M_LiFSI in DME	1.5	6
No. 9	NMC811	4M_LiFSI in DME	3	0.6
No. 10	NMC811	4M_LiFSI in DME	3	0.6
No. 11	NMC811	4M_LiFSI in DME	3	1.5
No. 12	NMC811	4M_LiFSI in DME	3	1.5

No. 13	NMC811	4M_LiFSI in DME	3	3
No. 14	NMC811	4M_LiFSI in DME	3	3
No. 15	NMC811	4M_LiFSI in DME	3	6
No. 16	NMC811	4M_LiFSI in DME	3	6
Unseen Batteries				
A	NMC811	4M_LiFSI in DME	0.6	1.5
B	NMC811	4M_LiFSI in DME	0.6	3
C	NMC811	4M_LiFSI in DME	0.6	6
D	NMC811	4M_LiFSI in DME	1.5	0.6

Supplementary Table S2. Frequency points for electrochemical impedance spectroscopy.

**Frequency range**	**Frequency points**
0 – 0.2 Hz	0.0093, 0.0139, 0.0209, 0.0326, 0.0488, 0.0721, 0.1071, 0.1583
0.2 – 2 Hz	0.2352, 0.3492, 0.5169, 0.7683, 1.141, 1.693
2 - 20 Hz	2.512, 3.728, 5.534, 8.121, 12.18, 18.08
20 – 200 Hz	26.82, 39.80, 59.06, 87.68, 130.1, 193.0
200 – 10 kHz	286.6, 425.1, 630.7, 936.1, 1390, 2063, 3061, 4542, 6740, 10000

Supplementary Table S3. Top 5 SHAP value features for the other testing batteries (refer to the supplementary Excel file for detailed numbering)

Cell	SFI-top5-features	SHAP values
4	63 52 64 51 54	0.091, 0.083, 0.061, 0.048, 0.046
6	63 64 52 51 53	0.100, 0.062, 0.033, 0.022, 0.022
8	63 64 36 59 52	0.167, 0.100, 0.024, 0.021, 0.018
10	52 63 28 51 53	0.123, 0.078, 0.062, 0.055, 0.053
12	63 64 52 53 51	0.104, 0.074, 0.040, 0.026, 0.024
14	63 64 52 62 36	0.142, 0.087, 0.020, 0.017, 0.016
16	63 64 62 52 36	0.167, 0.097, 0.021, 0.020, 0.019

Supplementary Table S4. The top 5 features with the highest overall SHAP values

O-SFI-top5-features	Summarized SHAP values
63	0.849
64	0.481
52	0.337
51	0.149
53	0.101

Supplementary Figures

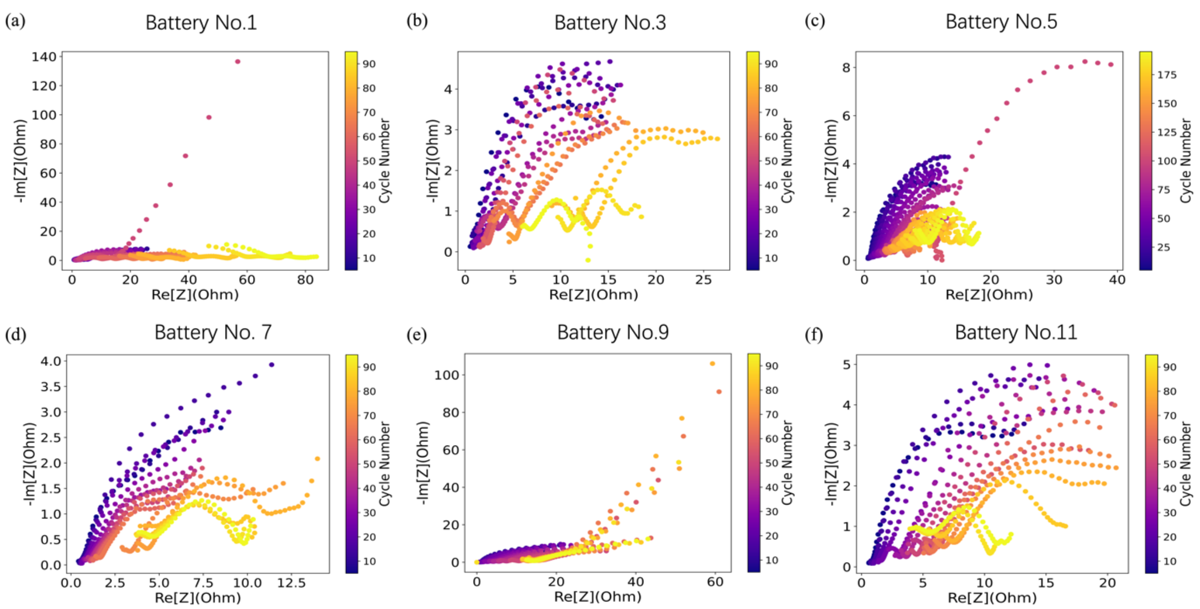

Figure S1. Impedance spectrum for the training batteries.

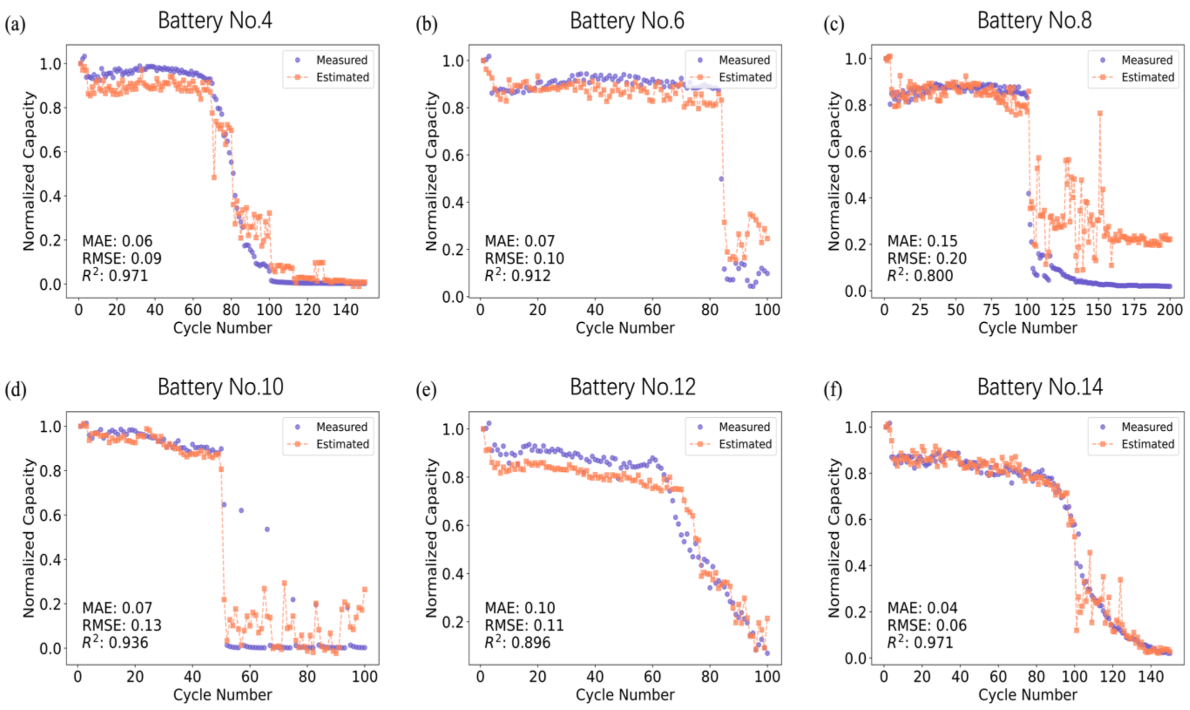

Figure S2. Estimated and Measured capacity as a function of cycle number for the remaining testing batteries in fully discharged state.

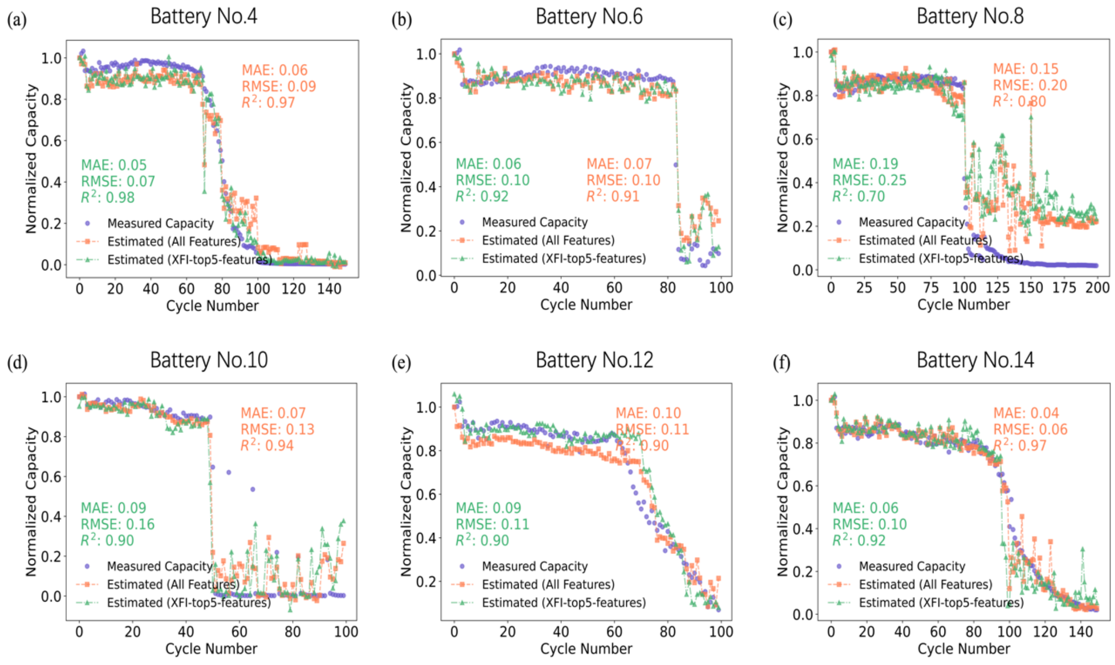

Figure S3. Comparison of the estimation results of the remaining testing batteries using the whole EIS data and XFI-top5-features.

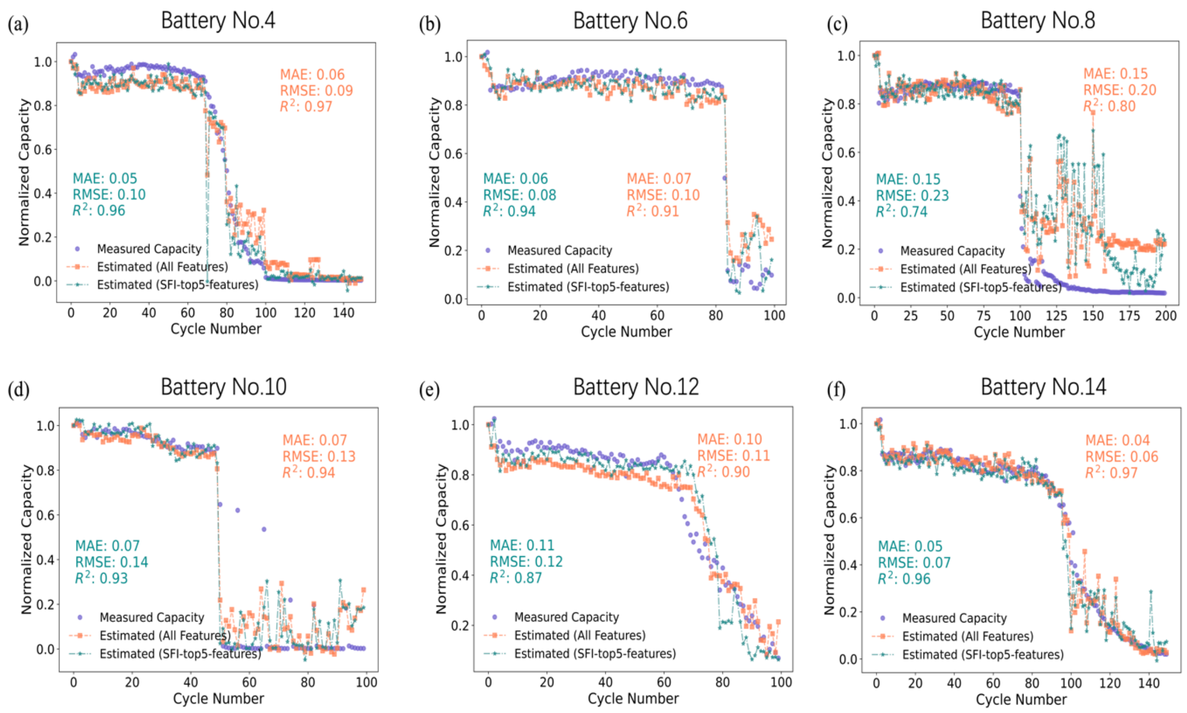

Figure S4. Comparison of the estimation results for the other testing batteries using the whole EIS dataset versus SFI-top5-features.

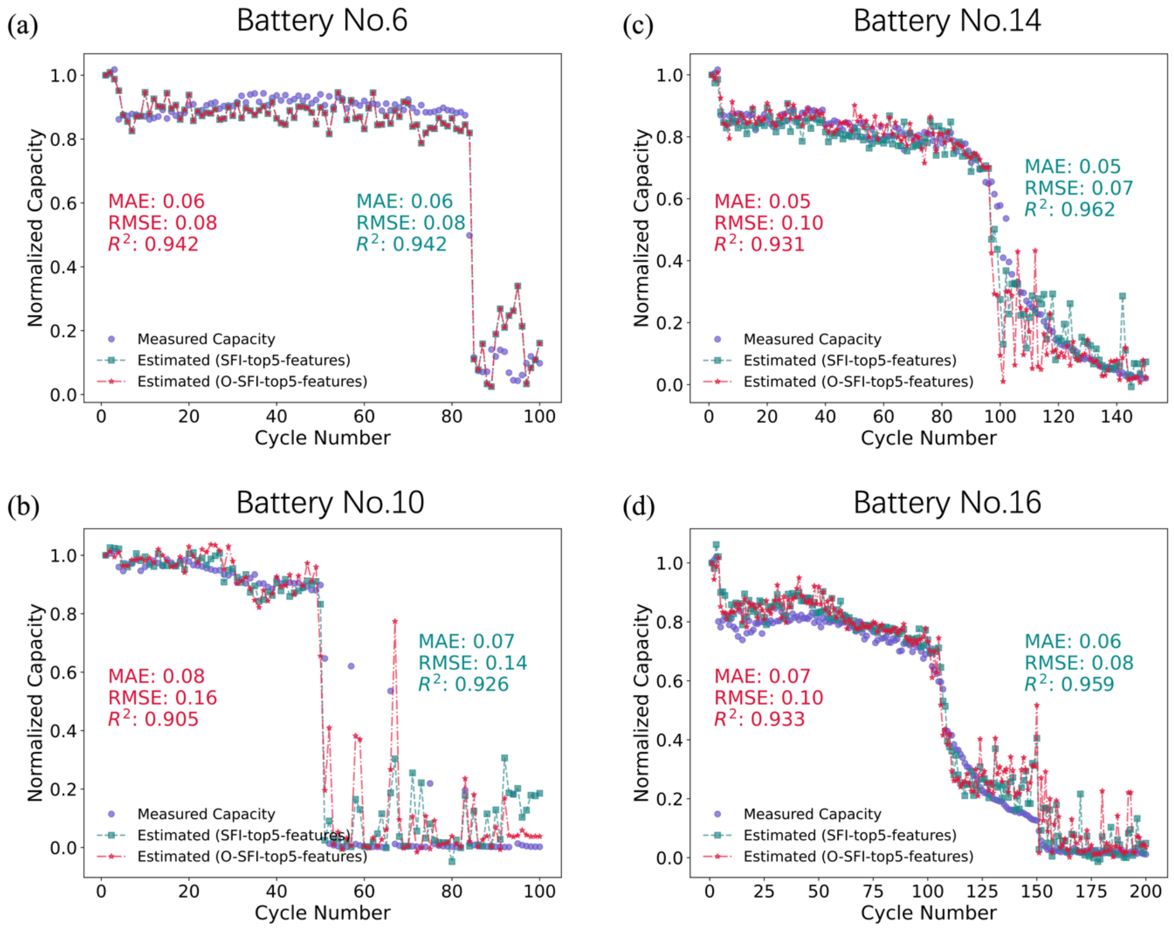

Figure S5. Comparison of the estimation results using SFI-top5-features and O-SFI-top5-features on four different testing batteries Battery No. 6, Battery No. 10, Battery No. 14 and Battery No.16.

Supplementary Note 1 XGBoost algorithm introduction

XGBoost is a highly flexible and efficient tree-boosting algorithm tailored for scalability, adaptability, and easy deployment. It integrates machine learning methods within the Gradient Boosting framework. Unlike multiple linear regression, XGBoost is particularly proficient in handling non-linear relationships. The mathematical formulation of the tree, represented as f(x), is as follows:

$$f_t(x) = \omega_{q(x)}, (q: R^d \rightarrow \{1,2,\dots,T\}, \omega \in R^T) \quad (1)$$

In this context, 't' represents a tree, 'q' refers to the structure of each tree that maps an instance to its corresponding leaf position, and 'T' denotes the total number of leaves in the tree. Each 'ft' is associated with a unique tree structure 'q' and leaf values 'ω' (which represent the result of the tree).

The objective function is:

$$obj^{(t)} = \sum_{i=1}^{n} l(y_i, \hat{y}_i^{(t)}) + \sum_{i=1}^{t} \Omega(f_i) \quad (2)$$

Here, 'l' represents a continuously differentiable convex loss function that measures the difference between the predicted value '$\hat{y}$' and the actual target 'y_i'. The second term, 'Ω', applies a penalty to the complexity of the model, helping to regularize the learned weights and reduce the risk of overfitting.

$$\Omega(f) = \gamma T + \frac{1}{2}\lambda \sum_{j=1}^{T} \omega_j^2 \quad (3)$$

In this equation, 'ωj' is the weight assigned to the 'j-th' leaf node. 'γ' and 'λ' control the magnitude of the penalty term 'Ω'.

$$obj^{(t)} = \sum_{i=1}^{n} l\left(y_i, \hat{y}_i^{(t-1)} + f_t(x_i)\right) + \Omega(f_t) + constant$$

$$\approx \sum_{i=1}^{n} \left(l\left(y_i, \hat{y}_i^{(t-1)}\right) + g_i f_t(x_i) + \frac{1}{2} h_i f_t^2(x_i)\right) + \Omega(f_t) + constant \quad (4)$$

Here, $g_i = \partial_{\hat{y}^{(t-1)}} l(y_i, \hat{y}^{(t-1)})$ and $h_i = \partial^2_{\hat{y}^{(t-1)}} l(y_i, \hat{y}^{(t-1)})$. Equation (5) is the objective function after removing the constant.

$$obj^{(t)}d = \sum_{i=1}^{(t)} \left(g_i \omega_{q(x_i)} + \frac{1}{2} h_i \omega^2_{q(x_i)}\right) + \gamma T + \frac{1}{2}\lambda \sum_{j=1}^{T} \omega_j^2 \tag{5}$$

$$= \sum_{j=1}^{T} \left(G_j \omega_j + \frac{1}{2}(H_j + \lambda)\omega_j^2\right) + \gamma T \tag{6}$$

Where $G_j = \sum\ i \in I_j g_i, H_j = \sum\ i \in I_j h_i, I_j = \{i|q(x_i = j)\}$.

The optimal weight 'ω_j^*' for the 'j-th' leaf node in a specific structure 'q(x)' can be determined using the following formula:

$$\omega_j^* = -\frac{G_j}{H_j + \lambda} \tag{7}$$

The optimal loss is:

$$obj^* = -\frac{1}{2}\sum_{j=1}^{T} \frac{(G_j)^2}{H_j + \lambda} + \gamma T \tag{8}$$

The function ' obj^*' is a measure of the quality of a tree structure and is dependent on the characteristics of the tree being evaluated. A lower value of ' obj^*' indicates a better or more favorable tree structure.

In the experiment, the following hyperparameters were adjusted to optimize the XGBoost model's performance:

Supplementary Note 2 Hyperparameter Tuning

n_estimators:
This parameter represents the number of boosting rounds or iterations the model goes through during training. Setting 'n_estimators' too low can lead to underfitting, meaning the model doesn't fully learn the underlying patterns in the data. On the other hand, setting it too high may lead to overfitting, where the model becomes too complex and learns noise in the data.

max_depth:
This parameter controls the maximum depth of each tree in the model. A higher 'max_depth' leads to more complex trees with stronger fitting capabilities, but it can also make the model

more prone to overfitting. Therefore, choosing an optimal depth is crucial for balancing complexity and generalization.

learning_rate:
The learning rate is an important parameter that dictates the step size the model takes during each iteration. A smaller learning rate helps make the model more robust, allowing it to converge more smoothly, though it may require more boosting rounds ('n_estimators') to reach optimal performance. A larger learning rate might lead to faster convergence but can risk overshooting the optimal solution.

subsample:
This parameter controls the fraction of training data used for building each tree. By setting a value between 0 and 1, it introduces randomness into the model, preventing overfitting by not using all data points for each tree. This can help improve generalization and reduce model variance.

colsample_bytree:
This parameter defines the fraction of features (columns) that are randomly sampled for constructing each tree. Like 'subsample', it introduces randomness and reduces the chance of overfitting, making the model more robust and capable of generalizing well to unseen data.

To fine-tune the hyperparameters of the EIS-Capacity model, a grid search strategy was implemented using the GridSearchCV function from the scikit-learn library. The search space was defined by specifying the following parameter ranges: n_estimators from [50, 100, 200, 300], max_depth from [3, 5, 7], learning_rate from [0.01, 0.1, 0.2], subsample from [0.6, 0.7, 0.8, 0.9], and colsample_bytree from [0.6, 0.7, 0.8, 0.9]. The grid search was conducted using 5-fold cross-validation, systematically evaluating all possible hyperparameter combinations. The performance of each configuration was measured using the negative mean squared error as the evaluation metric. Through iterative training and validation, the optimal hyperparameters were identified as colsample_bytree: 0.8, learning_rate: 0.1, max_depth: 5, n_estimators: 300, and subsample: 0.9. These parameters were subsequently used to train the EIS-Capacity model.

For the EIS-KP model, the same grid search strategy was employed. The search space was defined with the following parameter ranges: n_estimators from [100, 200, 300], max_depth from [3, 5, 7], learning_rate from [0.01, 0.1, 0.2], subsample from [0.8, 0.9, 1.0], and colsample_bytree from [0.8, 0.9, 1.0]. The 5-fold cross-validation was also implemented, the performance of each configuration was measured using the mean absolute error as the evaluation metric. After an exhaustive search, the optimal hyperparameters for the EIS-KP model were determined as colsample_bytree: 0.9, learning_rate: 0.2, max_depth: 5, n_estimators: 200, and subsample: 1.0. These hyperparameters were used to train the final EIS-KP model.
